# Anal sphincter function in rectal intussusception and high and low “take‐off” external rectal prolapse—A prospective observational study

**DOI:** 10.1111/codi.17191

**Published:** 2024-10-06

**Authors:** Alexander O'Connor, Caroline M. Byrne, Nick Heywood, Matthew Davenport, Niels Klarskov, Abhiram Sharma, Edward Kiff, Karen Telford

**Affiliations:** ^1^ Faculty of Biology, Medicine, and Health The University of Manchester Manchester UK; ^2^ Department of Colorectal Surgery Manchester University NHS Foundation Trust Manchester UK; ^3^ Department of Colorectal Surgery East Lancashire NHS Hospitals Trust Blackburn UK; ^4^ Department of Obstetrics and Gynaecology Herlev University Hospital Herlev Denmark; ^5^ Faculty of Health and Medical Sciences University of Copenhagen Copenhagen Denmark

**Keywords:** anal acoustic reflectometry, anal manometry, faecal incontinence, rectal intussusception, rectal prolapse

## Abstract

**Aim:**

Rectal intussusception (RI) and external rectal prolapse (ERP) are associated with anal sphincter dysfunction. The aim of this study was to examine sphincter function with anal acoustic reflectometry (AAR) in RI and two distinct phenotypes of ERP termed high and low “take‐off”.

**Methods:**

A prospective study of patients with RI and ERP attending a tertiary pelvic floor unit. Clinical data, AAR, and conventional anal manometry were analysed according to the Oxford prolapse grade.

**Results:**

A total of 108 (102 [94%] female, median age 62 years [range: 26–95]) patients were recruited into three groups according to prolapse grade: Oxford grades I and II (intrarectal RI, *n* = 34), Oxford grades III and IV (intra‐anal RI, *n* = 35) and Oxford grade V (ERP, *n* = 39). As the grade of prolapse increased, resting AAR measurements of opening pressure, opening elastance, closing pressure, and closing elastance decreased (*p* < 0.001). Maximum resting pressure with manometry was reduced in ERP and intra‐anal RI compared to intrarectal RI (*p* < 0.001). However, incremental squeeze function was not different between the three groups with either AAR or manometry (*p* > 0.05).

There were no differences in AAR or manometry variables between grade IV RI (*n* = 18) and high take‐off ERP (*n* = 20) (*p* > 0.05). By contrast, opening pressure (*p* = 0.010), closing pressure (*p* = 0.019) and elastance (*p* = 0.022) were reduced in low take‐off ERP (*n* = 19).

**Conclusion:**

Increasing rectal prolapse grade is associated with reduced anal sphincter function at rest indicating internal anal sphincter dysfunction. Physiological differences exist between high and low take‐off ERP with sphincter function in the former similar to that seen in grade IV RI.

**What does this paper add to the literature?**

The pathophysiology of rectal intussusception and rectal prolapse is unclear and may represent one progressive disease. This study identified an association between internal anal sphincter dysfunction and the grade of prolapse independent of age. Additionally, two distinct phenotypes of external prolapse are described which may have implications for surgical practice.

## INTRODUCTION

Rectal intussusception (RI), or internal rectal prolapse, is telescoping of the rectal wall inferiorly during defaecation [1], whilst external rectal prolapse (ERP) is full thickness protrusion of the rectum beyond the anal verge [2]. It has been suggested that RI is the beginning of a disease process that will progress to ERP over time. However, the exact pathophysiology of these conditions is not fully understood and the limited data available in retrospective series suggest progression from RI to ERP is rare [3, 4]. It has also been suggested that the level of the lead point, or “take‐off”, is an important clinical feature of ERP. The distinction between high and low “take off” may be relevant in the understanding of the condition and the outcomes following corrective surgical repair [5, 6].

RI is classified according to the lowest extent of the intussusception using the Oxford prolapse grading system on defaecating proctography (DP) [7]. Whilst RI may represent a normal phenomenon in up to 22% of asymptomatic volunteers [8, 9], it is also found in approximately 30% of patients with symptoms of disordered defaecation [10]. RI and ERP can present with obstructed defaecation syndrome (ODS), faecal incontinence (FI), anorectal pain and rectal bleeding causing a significant negative impact on quality of life (QoL) [11].

Using anorectal manometry in symptomatic patients, authors have sought to understand the pathophysiology underpinning RI and ERP with mixed results. This has contributed to the challenges in defining the natural history of these conditions and predicting successful treatment options which are frequently unsatisfactory with poor functional outcomes and high recurrence rates [12, 13]. In contrast to the fixed‐diameter anal manometry devices, anal acoustic reflectometry (AAR) is a dynamic test of anal sphincter function using volume‐controlled distension [14, 15]. It assesses the length‐tension relationship of the sphincter muscle which has been proposed as the key mechanism to describe sphincter dysfunction [16, 17].

It is hypothesized that there may be a decrease in anal sphincter function associated with higher grades of RI and ERP. Therefore, this prospective study aimed to investigate anal sphincter function, measured with AAR, and conventional anal manometry (AM), between the Oxford grades of RI and ERP. In addition, we describe two distinct phenotypes of ERP seen in clinical practice termed high and low take‐off ERP and examined the AAR and AM measurements in these groups.

## METHODS

### Participants

This prospective observational study recruited symptomatic adult patients attending a tertiary colorectal pelvic floor unit with functional anorectal disorders including FI, ODS and rectal prolapse. All patients were assessed by a consultant colorectal surgeon who recorded a full history and performed a clinical examination. Patients were identified as having RI or ERP on DP interpreted by a consultant gastrointestinal radiologist or, in the case of ERP, on clinical examination alone. Patients with primary or recurrent ERP were both included in this study. Patients were excluded if they lacked cognitive capacity to consent to the study, or if they had a diagnosis of inflammatory bowel disease or anorectal cancer. The DP findings of included patients were routinely discussed at the pelvic floor multidisciplinary team meeting and the RI graded according to the Oxford prolapse grading system [7]. Patients were then stratified into one of three groups according to the prolapse grade:

**Table:** 

Intrarectal RI (low‐grade RI)	Oxford grades I (high rectal) and II (low rectal)
Intra‐anal RI (high‐grade RI)	Oxford grades III (high anal) and IV (low anal)
External rectal prolapse	Oxford grade V (overt rectal prolapse)

### High and low take‐off external rectal prolapse

**FIGURE 1:**
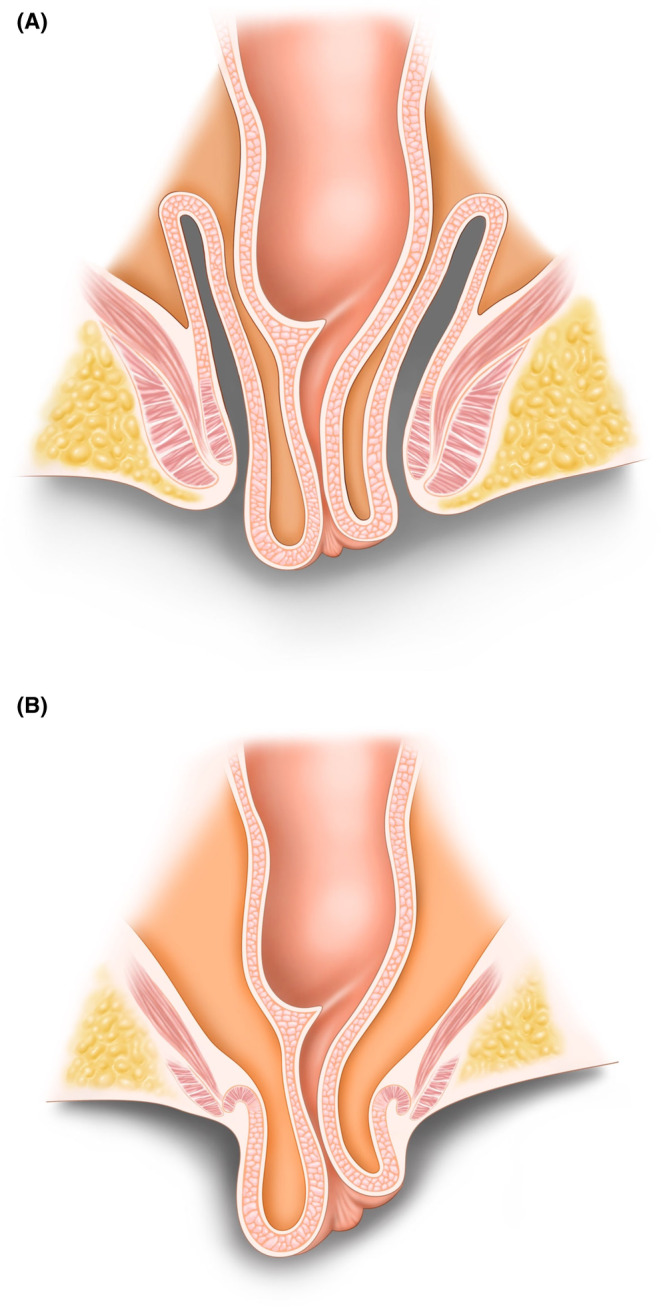
High take‐off (A) and low take‐off (B) external rectal prolapse.

In our unit it is routine practice to describe the anatomy of ERP according to the level of “take‐off” which has implications for the operative strategy. In high take‐off ERP the lead point of the prolapse arises from the mid‐ to low rectum above the anal sphincter complex and protrudes through the anal canal beyond the anal verge. A palpable sulcus can be felt between the prolapsing rectum and the anal canal (Figure 1A). In low take‐off ERP there is evagination of the sphincter complex and the lower rectum resulting in a visible external rectal prolapse. In such cases, there is no palpable sulcus, and the perianal skin becomes continuous with the anal and then rectal mucosa which is exteriorized (Figure 1B). Assessment of the “take‐off” level of the included ERP patients occurred independently of the patient recruitment to this study and was performed by a consultant colorectal and pelvic floor surgeon. To explore differences between these phenotypes a comparison was performed between patients with Oxford grade IV and high and low take‐off ERP.

### Evaluation

Patients provided written informed consent before demographic, symptom, obstetric and surgical data were recorded. The presence and pattern of FI (urge, passive or mixed) was recorded along with symptoms of urinary incontinence, faecal leakage and ODS which included the perceived difficulty in evacuation, a sensation of incomplete emptying, straining or regular digitation. Details of previous obstetric, gynaecological, anorectal and abdominal surgery were also recorded. Patients then underwent assessment with AAR followed by conventional AM.

FI and constipation severity was assessed using the St Mark's Incontinence Score (SMIS) [18] and the Constipation Scoring System (CSS) [19], respectively. QoL was assessed with the Manchester Health Questionnaire (MHQ) which assesses QoL impact across nine domains (overall health perception, impact on life, physical limitation, social limitation, relationship impact, emotional impact, sleep and energy impact and overall severity) [20]. A score of 0 represents no impact on QoL and a maximum score of 900 represents the greatest impact.

### Anal acoustic reflectometry

The technique of AAR and the measured variables have been described previously [15, 21]. Briefly, a highly compliant polyurethane bag of negligible cross‐sectional area (CSA) (0.4 mm^2^) is placed inside the anal canal. A digital signal processor (ED‐1932; Knowles Electronics, IL, USA) transmits wide‐band sound waves into the bag with the reflected acoustic impulses used to calculate the CSA [22] whilst pressure is simultaneously recorded by a transducer (SX30D, Sensym sensor systems). During assessment at rest, the bag is fully inflated with air over 7 s before being deflated over a further 7 s. A total of 10 cycles of inflation and deflation are performed at rest. Following this, assessment of voluntary squeeze is performed where the patient is asked to squeeze whilst the bag is inflated, a process repeated five times.

**FIGURE 2:**
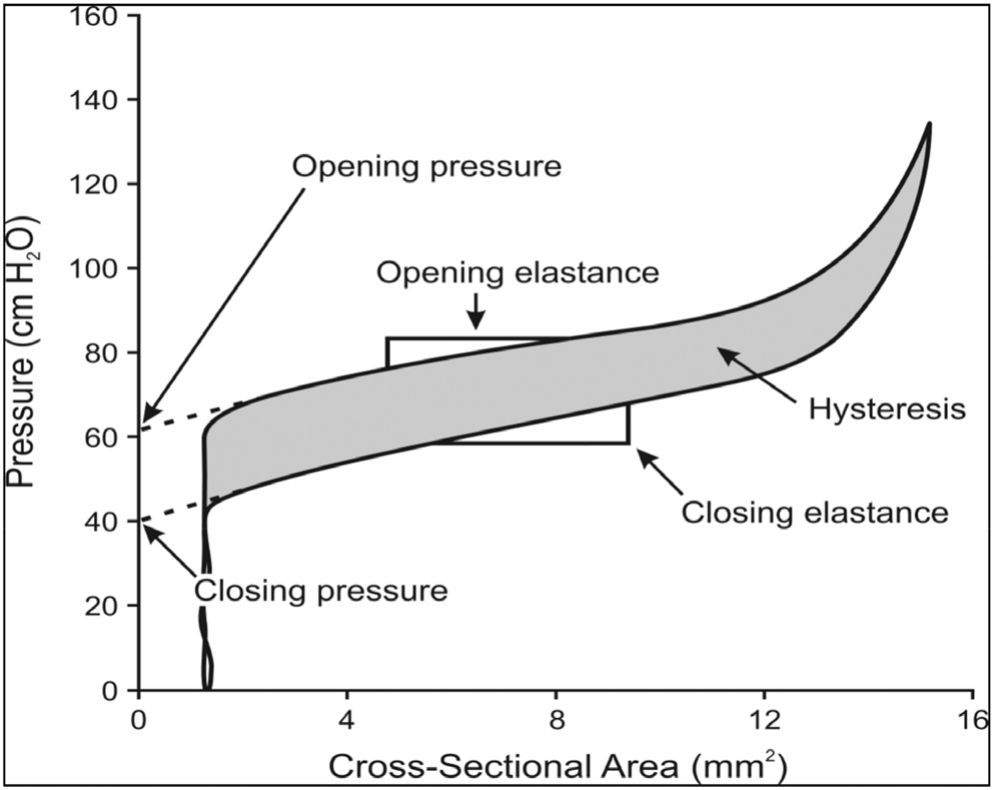
A graph of cross‐sectional area versus pressure after one cycle of inflation and deflation with anal acoustic reflectometry. (reproduced with permission from Mitchell et al. [43] © Wolters Kluwer Health Inc. Publishing licence no. 5380741033645).

The resulting measurements are used to create a graph of CSA versus pressure to calculate AAR variables at the point of the minimum CSA. At rest, five parameters are observed including the opening pressure (Op, cmH_2_O) which reflect the pressure the anal canal starts to open during distension. The closing pressure (Cp, cmH_2_O) is the pressure the anal canal closes down again following an episode of distension. Opening elastance (Oe, cmH_2_O/mm^2^), and closing elastance (Ce, cmH_2_O/mm^2^) reflect the resistance of the anal canal to distension, or the resistance to it closing, respectively. Hysteresis (Hys, %) reflects the amount of energy dissipated during one cycle of opening and closing of the anal canal. Two parameters are calculated during voluntary contraction: squeeze opening pressure (SqOp, cmH_2_O), and squeeze opening elastance (SqOe, cmH_2_O/mm^2^). Figure 2 presents a graph of CSA versus pressure obtained from one cycle of opening and closing (inflation and deflation) of the AAR bag in anal canal at rest with the five resting parameters highlighted. The difference between SqOp and Op is defined as the incremental squeeze opening pressure (IncSqOp, cmH_2_O) and reflects the maximum pressure generated by striated muscle during squeeze above the Op at rest. These measured parameters and their clinical significance have been discussed previously [23, 24].

### Conventional anal manometry

Conventional anal manometry (AM) was performed a minimum of 2 min following AAR. A manual station pull‐through technique was used with an in‐house manufactured closed water‐filled micro‐balloon as previously described [25]. Resting and squeeze pressures were recorded at each centimetre beginning at 5 cm proximal to the anal verge. Maximum resting pressure (MRP) and maximum squeeze pressure (MSP) were recorded relative to atmospheric pressure in cmH_2_O. Incremental squeeze pressure (ISP) was calculated as the difference between MSP and MRP in cmH_2_O.

### Power calculation

AAR has not previously been used in the investigation of RI or ERP. Therefore, an effect size of 0.8 was considered to represent the minimum clinically significant difference in opening pressure between groups (8 cmH_2_O). Patients were in three groups with an expected difference between each. With an 80% power and a two‐tailed 2% significance level to account for multiple testing, 33 patients were required in each group of intrarectal RI (Oxford grades I–II), intra‐anal RI (Oxford grades III–IV) and ERP (Oxford grade V).

### Statistical analysis

Statistical analysis was performed with SPSS for Mac (version 29.0, IBM, NY, USA). Continuous data are reported as median (interquartile range [IQR]), unless otherwise stated. The Kruskal‐Wallis and Mann–Whitney U tests were used for comparisons between independent samples of nonparametric data. Any post hoc pairwise comparisons between two groups were performed with statistical significance adjusted by the Bonferroni correction for multiple tests. Categorical variables are reported as *N* (percentage) with chi‐squared tests to compare the categorical variables. Statistical significance was considered at *p* < 0.05 level.

## RESULTS

Between January 2016 and June 2023, a total of 108 patients (102 [94%] female, median age 62 years [range: 26–95]) were prospectively recruited into the three groups: intrarectal RI (*n* = 34), intra‐anal RI (*n* = 35) and ERP (*n* = 39). Baseline demographic, symptomatology and past obstetric and surgical data are presented in Table 1.

**TABLE 1:** Baseline patient data.

Variable	Intra‐rectal RI (*n* = 34)	Intra‐anal RI (*n* = 35)	ERP (*n* = 39)	*p*‐value^f^
Age, median (range)	55 (32–77)	65 (40–83)	64 (26–95)	**0.014**
Male/female, *n* (%)	2 (6%)/32 (94%)	2 (6%)/33 (94%)	2 (5%)/37 (95%)	0.989
Weight (kg)	76.2 (66.7–92.1)	70.0 (64.5–82.7)	68.5 (62.5–77.0)	0.077
Obstetric history, *n* (%)				
Parous^a^	30/32 (94%)	30/33 (91%)	26/37 (70%)	**0.013**
Vaginal delivery^b^	29/30 (97%)	28/30 (93%)	24/26 (92%)	0.762
Caesarean section^b^	7/30 (23%)	3/30 (10%)	3/26 (12%)	0.294
Forceps use^c^	12/29 (41%)	9/28 (32%)	4/24 (17%)	0.150
Ventouse use^c^	0/29 (0%)	1/28 (4%)	0/24 (0%)	0.384
Episiotomies or perineal tear^c^	27/29 (93%)	21/28 (75%)	15/24 (63%)	**0.026**
Urinary incontinence, *n* (%)	12 (35%)	13 (37%)	12 (31%)	0.387
Bowel function history, *n* (%)				
Obstructed defecation syndrome	22 (65%)	24 (69%)	29 (74%)	0.665
Faecal incontinence	21 (62%)	30 (86%)	34 (87%)	**0.014**
Urge faecal incontinence^d^	10/21 (48%)	4/30 (13%)	6/34 (18%)	**0.010**
Passive faecal incontinence^d^	2/21 (10%)	9/30 (30%)	10/34 (29%)	0.177
Mixed faecal incontinence^d^	9/21 (43%)	17/30 (57%)	18/34 (53%)	0.614
Faecal leakage	15 (44%)	27 (77%)	31 (79%)	**0.002**
Previous gynaecological surgery, *n* (%)^a^	20/32 (63%)	21/33 (64%)	21/37 (57%)	0.817
Hysterectomy^e^	9	11	14	
Anterior repair^e^	4	2	1	
Sterilization^e^	1	2	4	
Other^e^	8	9	1	
Previous anorectal surgery, *n* (%)	8 (24%)	7 (20%)	14 (36%)	0.265
Rectocele repair^e^	5	5	1	
Haemorrhoidectomy^e^	3	2	2	
Perineal prolapse repair^e^	0	0	13	
Other^e^	3	2	0	
Previous abdominal surgery, *n* (%)	19 (55%)	12 (34%)	16 (41%)	0.180
Appendicectomy^e^	5	7	8	
Cholecystectomy^e^	7	4	3	
Abdominal rectopexy^e^	0	0	8	
Other^e^	4	2	1	

Abbreviations: ERP, external rectal prolapse; RI, rectal intussusception.

^a^ Females only.

^b^ Percentage calculated from parous females only.

^c^ Percentage calculated from females with vaginal deliveries only.

^d^ Percentage calculated from patients with faecal incontinence.

^e^ Number of procedures.

^f^ Chi‐squared test (age and weight examined with Kruskal–Wallis test). Significance at *p* < 0.05 level.

Patients with intrarectal RI were younger in age than those with intra‐anal RI (55 vs. 65; adjusted *p*‐value = 0.023) but there was no difference in age between patients with intra‐anal RI and patients with ERP (65 vs. 64; adjusted *p*‐value = 1.00). There were fewer parous females in the ERP group compared to the intrarectal and intra‐anal RI cohorts (*p* = 0.003) although, of all parous females in the study, the majority had undergone a vaginal delivery (81/86, 94%) and reported perineal trauma (63/81, 78%). There was no difference in the proportion of patients with ODS symptoms across the groups; however, FI and faecal leakage were more frequently reported in the ERP group (*p* = 0.011 and *p* = 0.002). There were no differences in SMIS, CSS or MHQ results between the three groups (Table 2).

**TABLE 2:** Patient reported symptom severity questionnaire results.

Variable	Intrarectal RI (*n* = 34)	Intra‐anal RI (*n* = 35)	ERP (*n* = 39)	*p*‐value^a^
St Mark's Incontinence Score	10 (2–18)	14 (10–17)	16 (14–18)	0.053
Constipation Scoring System	9 (4–13)	9 (5–13)	12 (7–17)	0.153
Manchester Health Questionnaire	454.16 (251.45–572.29)	439.17 (251.67–599.17)	530.00 (420.83–613.34)	0.079

Abbreviations: ERP, external rectal prolapse; RI, rectal intussusception.

^a^ Kruskal–Wallis test. Significance at *p* < 0.05 level.

In the ERP group, 19 (19/39, 49%) patients presented with recurrent ERP having previously undergone a total of 21 surgical procedures for rectal prolapse. However, with the exception of SqOe (*p* = 0.035) there were no differences in age, AAR, AM or symptom severity measures between those with primary or recurrent ERP (Table S1).

### Comparison of AAR and conventional AM between groups

**FIGURE 3:**
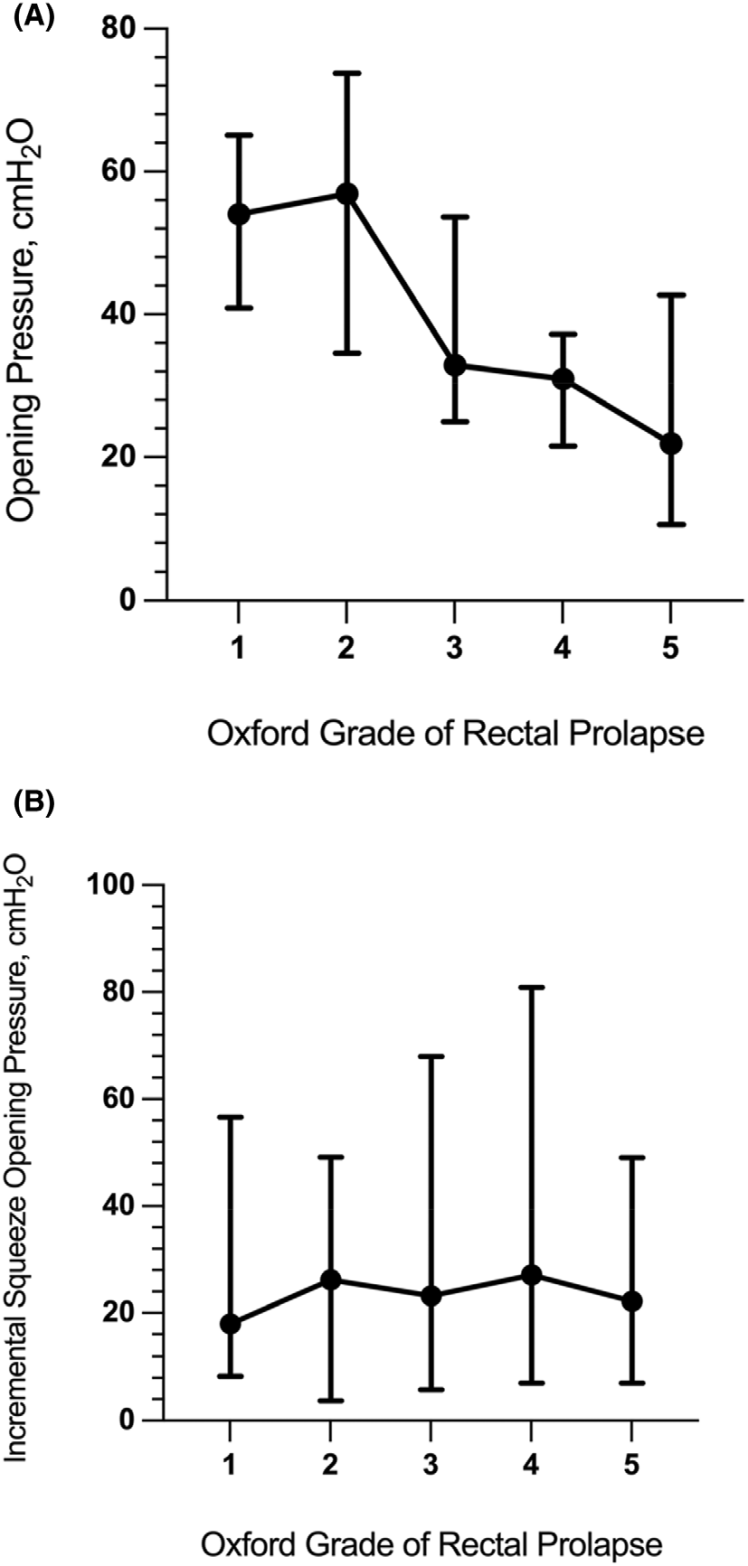
Graphs demonstrating the median and interquartile range of opening pressure (A) and incremental squeeze opening pressure (B) according to the Oxford Grading System.

As the grade of prolapse increased from intrarectal RI to ERP, a significant decrease was identified in the resting AAR parameters of Op, Oe, Cp and Ce (*p* < 0.001 for each); however, no significant difference was seen in hysteresis between groups (Table 3). MRP was reduced in ERP and intra‐anal RI compared to intrarectal RI (*p* < 0.001). There were no significant differences in absolute SqOp or incremental SqOp between the groups. Figure 3 demonstrates the differences in Op and IncSqOp between the five groups of the Oxford grading system and highlights the decline in Op whilst IncSqOp remained similar between groups. Whilst MSP was reduced in ERP and intra‐anal RI compared to intrarectal RI (*p* = 0.025), there was no difference in ISP.

**TABLE 3:** Anal acoustic reflectometry and conventional anal manometry results according to the grade of rectal intussusception or external rectal prolapse.

Parameter	Intrarectal RI (*n* = 34)	Intra‐anal RI (*n* = 35)	ERP (*n* = 39)	*p*‐value^a^
Anal acoustic reflectometry				
Opening pressure (Op), cmH_2_O	55.4 (38.0–66.3)	31.5 (24.5–49.4)	21.9 (10.6–42.7)	**<0.001**
Opening elastance (Oe), cmH_2_O/mm^2^	2.0 (1.4–2.4)	1.4 (1.0–1.7)	1.1 (0.8–1.3)	**<0.001**
Closing pressure (Cp), cmH_2_O	26.4 (20.3–37.1)	17.7 (11.1–28.2)	10.5 (3.7–26.8)	**<0.001**
Closing elastance (Ce), cmH_2_O/mm^2^	1.6 (1.2–2.1)	1.1 (0.9–1.4)	0.8 (0.6–1.1)	**<0.001**
Hysteresis (Hys), %	39.9 (34.3–47.9)	41.2 (36.0–47.1)	45.1 (32.7–54.0)	0.504
Squeeze opening pressure (SqOp), cmH_2_O	74.8 (47.7–113.7)	76.5 (37.1–110.1)	57.0 (23.2–92.5)	0.060
Squeeze opening elastance (SqOe), cmH_2_O/mm^2^	1.7 (1.4–2.1)	1.2 (1.0–2.0)	1.3 (0.9–1.8)	**0.017**
Incremental squeeze opening pressure (IncSqOp), cmH_2_O	22.0 (5.2–50.0)	23.6 (7.1–71.4)	22.2 (7.1–49.0)	0.609
Conventional anal manometry				
Maximum resting pressure (MRP), cmH_2_O	61.5 (36.3–79.8)	22.0 (15.0–36.0)	29.0 (17.0–39.0)	**<0.001**
Maximum squeeze pressure (MSP), cmH_2_O	85.0 (53.5–127.3)	59.0 (25.0–98.0)	53.0 (30.0–82.0)	**0.023**
Incremental squeeze pressure, cmH_2_O	26.0 (6.3–49.0)	29.0 (5.0–62.0)	20.0 (10.0–39.0)	0.788

Abbreviations: ERP, external rectal prolapse; RI, rectal intussusception.

^a^ Kruskal‐Wallis test. Significance at *p* < 0.05 level.

Given that the age difference between patients with intrarectal RI and intra‐anal RI (55 vs. 65; adjusted *p*‐value = 0.023) may contribute to the decrease in resting AAR parameters, patients in both RI groups were age (±1 year) and sex matched with the results presented in Table S2. Similar differences were seen in Op, Oe, Cp, Ce and MRP (*p* < 0.001 for all).

### Comparison of grade IV RI and high and low take‐off ERP

Of the 39 patients with ERP, there were 20 and 19 identified with high and low take‐off ERP, respectively. These two groups were compared to patients with grade IV RI (*n* = 18) and the results are presented in Table 4. There were significant differences in the resting parameters of Op (*p* = 0.010), Cp (*p* = 0.019) and Ce (*p* = 0.022) between the groups with a decrease seen from grade IV to low take‐off ERP. Patients with low take‐off ERP were older than those with grade IV RI and high take‐off ERP (*p* = 0.011). Whilst SqOp (*p* = 0.008) and MSP (*p* = 0.038) were different between the groups, there was no difference in IncSqOp and ISP (*p* > 0.05 for both). When the results from grade IV RI were compared to high take‐off ERP in a pairwise comparison there were no differences in any measurement of resting or squeeze anal sphincter function (adjusted *p*‐value >0.05) with only CSS scores greater in the high take‐off ERP group (14.0 vs. 8.0; adjusted *p*‐value = 0.046).

**TABLE 4:** A comparison of anal acoustic reflectometry and conventional anal manometry parameters between grade IV rectal intussusception and high and low external rectal prolapse.

Parameter	Grade 4 RI (*n* = 18)	High take off ERP (*n* = 20)	Low take off ERP (*n* = 19)	*p*‐value^a^ (three groups)	Grade 4 versus high take‐off ERP adj *p* value^b^
Age, median (range)	66 (50–83)	62 (26–78)	79 (31–95)	**0.011**	0.399
Anal acoustic reflectometry					
Opening pressure (Op), cmH_2_O	31.0 (21.5–37.2)	30.6 (17.3–54.9)	13.9 (9.2–28.1)	**0.010**	1.000
Opening elastance (Oe), cmH_2_O/mm^2^	1.2 (1.0–1.7)	1.1 (0.8–1.6)	0.9 (0.6–1.2)	0.088	1.000
Closing pressure (Cp), cmH_2_O	18.1 (10.1–20.3)	18.2 (6.8–30.5)	4.9 (3.3–14.0)	**0.019**	1.000
Closing elastance (Ce), cmH_2_O/mm^2^	1.0 (0.8–1.3)	1.0 (0.7–1.2)	0.7 (0.5–0.8)	**0.022**	1.000
Hysteresis (Hys), %	42.2 (36.0–47.9)	39.0 (31.7–49.9)	48.5 (44.9–57.1)	0.135	1.000
Squeeze opening pressure (SqOp), cmH_2_O	69.7 (39.1–110.1)	84.4 (51.4–113.2)	42.7 (15.1–58.4)	**0.008**	1.000
Squeeze opening elastance (SqOe), cmH_2_O/mm^2^	1.1 (0.9–1.8)	1.4 (0.8–2.7)	1.2 (1.0–1.6)	0.569	1.000
Incremental squeeze opening pressure (IncSqOp), cmH_2_O	27.1 (7.1–80.9)	39.6 (10.3–85.5)	18.0 (5.3–41.4)	0.146	1.000
Conventional anal manometry					
Maximum resting pressure (MRP), cmH_2_O	24.0 (15.0–33.8)	31.0 (21.8–59.5)	23.0 (13.0–32.5)	0.155	0.490
Maximum squeeze pressure (MSP), cmH_2_O	54.0 (23.8–99.3)	70.5 (36.5–115.3)	42.0 (24.0–54.5)	**0.038**	0.705
Incremental squeeze pressure, cmH_2_O	29.5 (4.0–62.8)	34.5 (14.5–52.0)	14.0 (4.5–30.5)	0.098	1.000
Symptom Severity Scores					
St Mark's Incontinence Score	16.0 (14.0–18.0)	15.0 (12.0–18.0)	16.0 (14.0–18.0)	0.723	1.000
Constipation Scoring System	8.0 (3.0–12.0)	14.0 (9.0–17.0)	9.0 (6.0–17.0)	0.051	**0.046**
Manchester Health Questionnaire	445.83 (338.75–535.42)	552.92 (485.63–612.09)	502.50 (364.17–618.33)	0.099	0.103

Abbreviations: ERP, external rectal prolapse; RI, rectal intussusception.

^a^ Kruskal‐Wallis test. Significance at *p* < 0.05.

^b^ Significance adjusted by the Bonferroni correction for multiple tests.

## DISCUSSION

This prospective study investigated the pathophysiology of RI and ERP in symptomatic patients using AAR, a unique test of the length‐tension relationship of the sphincter muscle. It identified a decrease in resting measurements as the Oxford grade of prolapse increases, whilst squeeze function appears preserved, a finding that appears independent of age. High and low take‐off ERP phenotypes are routinely defined in our unit. This study identified differences between these ERP phenotypes and Oxford grade IV RI, with reduced sphincter function observed in the low take‐off ERP group. However, there was no difference in AAR and AM variables between grade IV RI and high take‐off ERP. This suggests these two conditions may be similar and that high take‐off ERP could represent a progression of RI below the anal verge. As in this study, previous work has typically grouped RI into low‐grade (I and II) and high‐grade (III and IV) according to the Oxford grade [7]; however, our results highlight how Oxford grade IV RI may best be compared to high take‐off ERP. Low take‐off ERP may therefore be a progression of high take‐off ERP with further weakening of the pelvic floor and rectal support structures, or it may represent a separate disease entity.

### Clinical implications

The two phenotypes of ERP described in this study could be considered separate conditions within the Oxford grade V category. This may have implications for the surgical strategy and risk of postoperative recurrence. Patients with high take‐off ERP may require an abdominal rectopexy for adequate fixation, whereas low take‐off ERP may be successfully managed with a perineal approach [6, 26]. If low take‐off ERP is indeed a progression of high take‐off prolapse then consideration should be made to the timing of surgery. Given low take‐off ERP is associated with the most deficient sphincter function, a delay in surgical correction could result in further sphincter damage and unsatisfactory functional outcomes. A deeper understanding of sphincter function measured with AAR could be used to guide the timing of surgery where a decrease in sphincter function is observed. Alternatively, AAR could inform the patient and clinician about expected continence symptoms following corrective surgical repair.

Surgical repair of ERP is considered the mainstay of treatment to ameliorate symptoms [2]. However, there remains uncertainty surrounding the optimum surgical strategy for symptomatic intra‐anal RI, with only poor‐quality heterogenous studies available [27]. In patients with unexplained incontinence, up to 63% can also have intra‐anal RI [7]; however, unsatisfactory functional outcomes and high recurrence rates are often reported following surgical correction [12, 28]. In our study, FI was the most common symptom in intra‐anal RI, and the majority reported either passive or mixed FI suggesting internal anal sphincter (IAS) dysfunction. This could explain the persistent FI experienced by up to 36% of patients following surgical correction of intra‐anal RI [28]. Careful consideration of surgical intervention and potential functional outcomes is therefore required. Our results suggest there are no differences in anal sphincter function between patients with grade IV RI and high take‐off ERP. Therefore, studies should now look to establish the role and timing of surgical treatment in these similar groups as it remains uncertain if sphincter function recovers after correction of the anatomical abnormality. Given the limited treatment options that currently exist for IAS dysfunction intervention early upon presentation with external prolapse may be necessary [29]. As there remains a suggestion this condition may progress over time, regular follow‐up with symptom assessment and AAR measurements may be warranted. This could be used to identify and prevent further progression of sphincter dysfunction with surgical and nonsurgical interventions.

### Natural history of rectal prolapse

The AAR parameter of opening pressure is the pressure at which the anal canal starts to open during distension, and is predominantly an assessment of IAS function [30]. The resistance to distension is measured with elastance, which is lower in patients with passive FI [24]. The findings in this study of reduced pressures and elastance at rest suggest an association between the grade of symptomatic prolapse and IAS dysfunction. This is further supported by the increased prevalence of FI and faecal leakage as the Oxford grade increases. Hysteresis reflects the amount of energy dissipated during one cycle of opening and closing the anal canal (Figure 2). It is greater in patients with FI compared to controls but is not associated with FI related to IAS dysfunction, and was no different between the three groups of RI and ERP in this study [25]. The IAS dysfunction seen in this study associated with the grade of prolapse may be a consequence of mechanical stress applied to the sphincter by the prolapsing rectum. Dvorkin et al. highlighted thickened and asymmetrical IAS on endoanal ultrasound in patients with ERP [31]. Indeed, the frequency of severe IAS injury reported on endoanal ultrasound is greater in patients with ERP compared to grade III/IV RI, and the severity of injury correlates with improvements in postoperative continence [32]. However other authors have suggested intra‐anal RI is associated with an absent or impaired rectoanal inhibitory reflex due to its persistent activation, or damage to the IAS resulting in symptoms of FI [33, 34]. Alternatively, the measured results may reflect a disorder or deficiency of collagen, which have been identified in patients with pelvic organ prolapse [35, 36]. There were no differences in incremental squeeze function between the grades of prolapse suggesting that striated muscle function may be preserved. The grade of prolapse therefore appears only associated with smooth muscle (IAS) dysfunction. However, the finding of comparable squeeze function in the ERP group may be influenced by the reduced number of parous females compared to the intrarectal and intra‐anal RI groups (*p* = 0.013).

Other authors have sought to establish a relationship between the Oxford grade of rectal prolapse and sphincter function measured with anorectal manometry. Retrospective work by Harmston et al. with 515 patients supports the findings of this study in demonstrating a reduction in MRP through the five Oxford grades of prolapse [37]. However, their study demonstrated a reduced maximal squeeze increment in the ERP cohort compared to those with grade IV RI (53.0 mmHg vs. 63.1 mmHg, *p* < 0.05), a finding not replicated in this study. Other authors have highlighted the association with MRP and the grade of prolapse, whilst also reporting unchanged squeeze function [38], or reduced MSP in high‐grade RI [39, 40] and ERP [33, 41].

RI has been suggested to be a progression of a disease which will lead to the development of ERP over time. In common with the study by Hotouras et al. [41], our study highlighted differences in age between the intrarectal RI and intra‐anal RI groups. However, when patients with RI were age (±1 year) and sex matched the same differences in sphincter function were observed as seen in the whole cohort. This indicates age may have a limited effect on the overall results. Wijffels et al. have proposed that a progression, should it exist, may occur at a rate of between 2.5 to 5.4 years/prolapse grade [42]. However, the correlation coefficients for parous females in their study (*r* = 0.41, *p* < 0.0001) and nulliparous females (*r* = 0.17, *p* = 0.16) were poor highlighting the evidence of disease progression on the basis of age alone is weak. Furthermore, the reported progression from intra‐anal RI to ERP remains low at approximately 4% in heterogenous studies with a median follow‐up of 5.6 years [3, 4, 41]. RI and ERP may therefore be unrelated, or progression occurs over a longer period, or the development of ERP is infrequent and only occurs as a consequence of several modifiable and nonmodifiable risk factors. In our study, sphincter dysfunction was no different between grade IV RI and high take‐off ERP suggesting the latter may represent a progression of RI beyond the anal verge. By contrast, low take‐off ERP may represent either further progression of the disease or is a separate condition occurring as a result of pelvic floor failure. Indeed, this group demonstrated the weakest sphincter function of all patients in this study. That these two distinct phenotypes seen in clinical practice are combined under the umbrella term of “Oxford grade V” may explain the mixed data presented in support of a single disease that progresses over time. Furthermore, if these conditions are indeed separate entities, the results of previous studies examining ERP surgical strategies, functional outcomes and recurrence rates may need to be reappraised. Consideration may need to be given to the mix of ERP phenotypes in these studies. Future work is therefore required to delineate the natural history of RI in long‐term observational studies and to elucidate the mechanistic pathways associated with disease progression.

This prospective study has highlighted an association between the Oxford grade of rectal prolapse and IAS dysfunction whilst describing different clinical phenotypes of ERP. Despite its relatively large size and prospective design there are limitations including the unequal distribution in parous females through the groups that may impact measurements of sphincter function. Approximately half (49%) of the ERP cohort were recruited to this study with recurrent ERP having undergone at least one previous surgical procedure for rectal prolapse. Whilst this reflects the typical patient cohort seen in a tertiary pelvic floor referral centre, it may have impacted the results of this study as it could be expected that these patients have the most deficient sphincter function. Finally, the single‐site design using a novel test alongside conventional AM limits the generalizability of the findings. Despite these limitations, this study has highlighted important mechanisms that may explain the pathophysiology underpinning symptomatic RI and ERP whilst highlighting future research needs.

## CONCLUSIONS

This study has highlighted as the Oxford grade of rectal prolapse increases, resting anal sphincter function deteriorates, whilst squeeze function appears preserved. This suggests an association with internal anal sphincter dysfunction and the grade of prolapse. Two distinct clinical phenotypes of ERP presented are termed high and low take‐off. These may represent a continuation of the same disease process, or separate conditions with the latter possibly reflecting failure of the pelvic floor. A deeper understanding of sphincter function with AAR could be used to guide the timing of surgical intervention or predict postoperative functional outcomes. Further work is now required to establish the natural history and mechanistic pathways associated with RI and ERP.

## AUTHOR CONTRIBUTIONS

**Alexander O'Connor:** Conceptualization; methodology; data curation; investigation; validation; formal analysis; funding acquisition; writing – original draft; project administration. **Caroline M. Byrne:** Conceptualization; methodology; data curation; investigation; validation; formal analysis; funding acquisition; writing – original draft; project administration. **Nick Heywood:** Conceptualization; methodology; data curation; validation; writing – review and editing. **Matthew Davenport:** Validation; formal analysis; writing – review and editing; visualization. **Niels Klarskov:** Methodology; software; supervision; investigation; writing – review and editing; resources. **Abhiram Sharma:** Conceptualization; supervision; writing – review and editing. **Edward Kiff:** Conceptualization; methodology; supervision; formal analysis; validation; writing – review and editing. **Karen Telford:** Supervision; writing – review and editing; resources; funding acquisition; project administration; conceptualization; methodology; visualization.

## FUNDING INFORMATION

A.O.C. and C.B. received funding from The Royal College of Surgeons of England.

## CONFLICT OF INTEREST STATEMENT

The authors declare no conflict of interests.

## ETHICS STATEMENT

The data presented in this manuscript were obtained from two studies which both received ethical approval from the local research ethics committee (Greater Manchester—East [Ref: 17/NW/0547] and Greater Manchester—Central [Ref: 16/NW/0033]).

## Supporting information


Table S1.

## Data Availability

The data presented in this manuscript are available from the corresponding author on reasonable request.
