# Supplementary material for: Anal sphincter function in rectal intussusception and high and low “take‐off” external rectal prolapse—A prospective observational study
Source: Colorectal Dis. 2024 Oct 6;26(12):2069–79. doi: 10.1111/codi.17191 (PMC11649865; doi:10.1111/codi.17191)
Supplement: Supplementary file 1 — Table S1. [file CODI-26-2069-s001.docx]

# Supplementary Tables

## Supplementary Table 1: Anal acoustic reflectometry, conventional anal manometry, and symptom severity results between patients recruited with primary or recurrent external rectal prolapse

| **Parameter** | **Primary ERP**  **(n = 20)** | **Recurrent ERP**  **(n = 19)** | **p value*** |
| --- | --- | --- | --- |
| Age, *median (range)* | 66  (47 – 80) | 64  (42 – 79) | 0.728 |
| **Anal Acoustic Reflectometry** | | | |
| Opening Pressure (Op), *cmH_2_0* | 27.9  (11.8 – 46.1) | 19.1  (7.7 – 30.2) | 0.204 |
| Opening Elastance (Oe), *cmH_2_0/mm^2^* | 1.1  (0.9 – 1.5) | 0.9  (0.7 – 1.2) | 0.247 |
| Closing Pressure (Cp), *cmH_2_0* | 11.0  (4.7 – 27.7) | 6.8  (2.5 – 21.2) | 0.380 |
| Closing Elastance (Ce), *cmH_2_0/mm^2^* | 0.8  (0.7 – 1.2) | 0.8  (0.4 – 0.9) | 0.141 |
| Hysteresis (Hys), *%* | 45.6  (33.8 – 53.1) | 45.1  (30.2 – 55.4) | 0.879 |
| Squeeze Opening Pressure (SqOp), *cmH_2_0* | 61.7  (28.8 – 85.6) | 55.3  (14.2 – 92.9) | 0.627 |
| Squeeze Opening Elastance (SqOe), *cmH_2_0/mm^2^* | 1.5  (1.0 – 2.6) | 1.1  (0.8 – 1.4) | **0.035** |
| Incremental Squeeze Opening Pressure (IncSqOp), *cmH_2_0* | 23.0  (10.8 – 43.6) | 22.2  (6.6 – 50.3) | 0.857 |
| **Conventional Anal Manometry** | | | |
| Maximum Resting Pressure (MRP), *cmH_2_0* | 30.0  (18.3 – 39.0) | 23.0  (16.5 – 48.0) | 0.858 |
| Maximum Squeeze Pressure (MSP), *cmH_2_0* | 56.5  (35.3 – 74.5) | 42.0  (30.0 – 105.0) | 0.883 |
| Incremental Squeeze Pressure, *cmH_2_0* | 22.5  (9.8 – 40.0) | 19.0  (10.5 – 45.5) | 0.858 |
| **Symptom Severity Scores** | | | |
| St Mark’s Incontinence Score | 16  (14 – 19) | 16  (13 – 18) | 0.923 |
| Constipation Scoring System | 12  (7 – 15) | 12  (6 – 19) | 0.708 |
| Manchester Health Questionnaire | 515.84  (394.38 – 606.34) | 547.50  (485.00 – 624.17) | 0.396 |
| ERP external rectal prolapse  *Mann-Whitney U Test. Significance at p < 0.05 level. | | | |

## Supplementary Table 2: Anal acoustic reflectometry, conventional anal manometry, and symptom severity results according to the grade of rectal intussusception or external rectal prolapse – RI patients matched by age (+/- 1 years)

| **Parameter** | **Intra-rectal RI**  **(n = 19)** | **Intra-anal RI**  **(n = 19)** | **ERP**  **(n = 39)** | **p value*** |
| --- | --- | --- | --- | --- |
| Age, *median (range)* | 59  (48 – 72) | 60  (48 – 73) | 64  (26 – 95) | 0.323 |
| **Anal Acoustic Reflectometry** | | | | |
| Opening Pressure (Op), *cmH_2_0* | 59.3  (43.5 – 65.1) | 37.1  (25.1 – 53.5) | 21.9  (10.6 – 42.7) | **<0.001** |
| Opening Elastance (Oe), *cmH_2_0/mm^2^* | 1.8  (1.5 – 2.4) | 1.4  (1.0 – 2.2) | 1.1  (0.8 – 1.3) | **<0.001** |
| Closing Pressure (Cp), *cmH_2_0* | 28.6  (23.2 – 36.0) | 20.6  (13.2 – 30.3) | 10.5  (3.7 – 26.8) | **<0.001** |
| Closing Elastance (Ce), *cmH_2_0/mm^2^* | 1.6  (1.3 – 2.1) | 1.2  (0.9 – 1.5) | 0.8  (0.6 – 1.1) | **<0.001** |
| Hysteresis (Hys), *%* | 37.4  (34.1 – 47.9) | 39.3  (35.9 – 47.1) | 45.1  (32.7 – 54.0) | 0.486 |
| Squeeze Opening Pressure (SqOp), *cmH_2_0* | 75.8  (47.7 – 106.0) | 66.2  (35.6 – 101.8) | 57.0  (23.2 – 92.5) | 0.171 |
| Squeeze Opening Elastance (SqOe), *cmH_2_0/mm^2^* | 1.8  (1.5 – 2.1) | 1.3  (0.9 – 1.9) | 1.3  (0.9 – 1.8) | **0.016** |
| Incremental Squeeze Opening Pressure (IncSqOp), *cmH_2_0* | 16.4  (5.6 – 49.1) | 16.1  (6.1 – 61.2) | 22.2  (7.1 – 49.0) | 0.668 |
| **Conventional Anal Manometry** | | | | |
| Maximum Resting Pressure (MRP), *cmH_2_0* | 56.5  (36.8 – 79.3) | 22.0  (15.0 – 36.0) | 29.0  (17.0 – 39.0) | **<0.001** |
| Maximum Squeeze Pressure (MSP), *cmH_2_0* | 81.5  (44.5 – 109.5) | 48.0  (27.0 – 98.0) | 53.0  (30.0 – 82.0) | 0.158 |
| Incremental Squeeze Pressure, *cmH_2_0* | 24.5  (4.8 – 42.8) | 22.0  (5.0 – 71.0) | 20.0  (10.0 – 39.0) | 0.918 |
| **Symptom Severity Scores** | | | | |
| St Mark’s Incontinence Score | 14  (2 – 20) | 14  (13 – 17) | 16  (14 – 18) | 0.586 |
| Constipation Scoring System | 10  (5 – 16) | 9  (7 – 13) | 12  (7 – 15) | 0.383 |
| Manchester Health Questionnaire | 520.83  (407.50 – 642.92) | 470.00  (306.67 – 645.00) | 530.00 (420.83 – 613.34) | 0.737 |
| ERP external rectal prolapse, RI rectal intussusception.  * Kruskal-Wallis test. Significance at p < 0.05 level. | | | | |
